# Knowledge, attitude and practice of surgical staff towards preoperative surgical antibiotic prophylaxis at an academic tertiary hospital in Sudan

**DOI:** 10.1186/s13037-019-0224-2

**Published:** 2019-12-11

**Authors:** Ali Mohammed Ahmed, Sara Nasr, Almegdad Mohamed Ahmed, Osama Elkhidir

**Affiliations:** 1 Faculty of Medicine, SAMER research group, Khartoum University, Alqaser street, 11111 Khartoum, Sudan; 2 Department of community medicine, Faculty of Medicine, Khartoum University, Alqaser street, 11111 Khartoum, Sudan

**Keywords:** Antibiotic prophylaxis, Knowledge, Attitude, Practice, Surgery, Surgeons

## Abstract

**Background:**

Surgical site infections (SSIs) are among the most common serious complications after surgery and associated with preventable morbidity, mortality, and increased health care costs. The use of surgical antimicrobial prophylaxis (SAP) is an effective measure that helps to protect against SSIs. This study aims to evaluate the knowledge, attitude, and practice of surgical staff towards preoperative antibiotic prophylaxis in surgery department at an academic tertiary hospital in Sudan.

**Methodology:**

An observational descriptive study was conducted among doctors in the surgery department at an academic tertiary hospital in Sudan in order to assess their knowledge, attitude, and practice (KAP) towards surgical antibiotic prophylaxis (SAP). A four-section multiple-choice questionnaire was designed and hand-delivered to registered doctors in the surgery department at an academic tertiary hospital in Sudan. The WHO guidelines were used to evaluate the answers of the participants.

**Results:**

Out of 56 doctors requested to participate in this study, only 49 responded and their response rate was 87.5%. Six (12.5%) surgeons had good knowledge about appropriate SAP. However, 16.3 and 24.5% of the respondents were aware of appropriate SAP in the case of Ig E-mediated reaction to penicillin and risk of Gram-negative infections, respectively. The surgeon’s attitude score about the need for local and national guidelines for SAP was 98 and 100%, respectively. Accordance of the physician’s practice with ASHP guidelines regarding timing of the first dosage of SAP was 35.4% while correct administration of an intraoperative dose was 42.9% in agreement with the guideline. 53.1% knows when to stop SAP after surgery correctly.

**Conclusion:**

Although the participants in this study showed a positive attitude towards antibiotic prophylaxis guidelines, their knowledge and strict adherence to a protocolized practice per WHO checklist should be improved in order to reduce the incidence of preventable surgical site infections.

## Introduction

Surgical site infections (SSIs) are one of the most common postoperative complications, affecting nearly half of the patients undergoing surgical procedures [1]. Several studies have identified SSIs as a cause for increased morbidity, mortality, length of hospital stay, health care costs [2–7]. Due to various factors including lack of resources and staff shortage, the problem of SSIs is more prominent in developing countries [8].

Surgical antibiotic prophylaxis (SAP) has been recognized as one of the major factors and essential tools in combating and decreasing SSIs [9–12]. Numerous guidelines have been developed, describing the types, dosage, and duration of administration of SAP [13–15]. However, several studies have demonstrated poor adherence among doctors towards these guidelines [16–18].

A study conducted in Sudan revealed that the prevalence of SSIs was 25% [19]. Although one study has concluded that there was a broad difference between the international guidelines and local practices in Sudan [20], there is a lack of sufficient evidence regarding doctors’ knowledge and practices towards SAP guidelines. In this study, the aim was to assess knowledge, attitude, and adherence to the practice of SAP guidelines among doctors at an academic tertiary hospital in Sudan.

## Materials and methods

This study was designed as a descriptive, facility-based cross-sectional study. Conducted at Soba University Hospital, which is the largest university hospital in the country, offering a wide variety of health services and educational training, with a capacity of 500 beds. All anesthesiology and surgery doctors, at all levels of training in the department of surgery at Soba University Hospital, were requested to participate in this study between December 2017 and January 2018.

The data regarding doctors’ knowledge, attitude and adherence to practice towards surgical antibiotic prophylaxis was collected using a self-administered, four-section, multiple-choice questionnaire that was designed, reviewed, and used by Baniasadi et all at their study [21]. In addition to the first part of the questionnaire, which included demographic data about the doctor’s age, gender, level of training, specialty, and level of training. It also has a second part, which was designed to assess doctors’ knowledge about the types of surgeries in which SAP is used in, the antibiotics of choices which are commonly used in different clinical scenarios, and their source of knowledge regarding SAP. It also includes a third party in which the participants’ attitude was evaluated towards preparation of national SAP guidelines, and their willingness to cooperate in the establishment of such guidelines. The last part of the questionnaire was dedicated to testing the adherence of doctors towards different SAP practices. Doctor’s answers were considered correct when they were in alignment with the ASHP guidelines, which were developed by the Society for Healthcare Epidemiology of America, the Surgical Infection Society, and the Infectious Diseases Society of America [14].

The data were entered and managed using SPSS v23. Numerical data were presented as means and standard deviations (SD) and categorical data as frequencies and percentages. Pearson’s correlation was used to test the correlation between participants’ knowledge and practice towards SAP. While spearman’s correlation was used to test the correlation between participants’ level of training, and their knowledge and practice towards SAP. A *P*-value < 0.05 was considered statistically significant.

This study was approved by the institutional review board at the department of community medicine in the faculty of medicine, University of Khartoum. Besides, written permission was sought from Soba University Hospital to conduct this study at their department. Moreover, consent was obtained from each respondent individually to participate in this study.

## Results

56 doctors were asked to participate in this study, of whom 49 (88%) responded to the questionnaire. More than half of the respondents 27 (55%) were female, and their mean age was 26 years. Moreover, 25 (51%) of the participants were specialty registrars at the time of the study, the vast majority of the 39 (80%) were surgical registrars, and with a mean of 2 years as clinical-based practice experience. Table 1 demonstrates the demographic characteristics of the participants.

**Table 1:** Demographic characteristic of the participants

Age mean (mean ± SD)	26.1 ± 2.4
Gender	N (%)
Male Female	22 (45) 27 (55)
Academic level	N (%)
House officer (foundation trainee)	22 (45)
Medical officer (senior house officer)	1 (2)
Specialty registrar	25 (51)
Specialist	1 (2)
Specialty	N (%)
Surgery	39 (80)
Anesthesiology	10 (20)
Years of experience (mean ± SD)	2 ± 1.5

Regarding the knowledge of the participants about the types of surgeries in which SAP is administered, 41 (84%) and 36 (74%) of the respondents answered correctly by saying that SAP is administered for clean surgeries that involve prosthesis and clean contaminated surgeries, respectively. However, a major portion of the 40 (82%) had chosen contaminated surgeries. Also, only 6 (13%) of the respondents had correctly selected the drug of choice for Gastro-duodenal surgeries which is Cefazolin, while nearly half of the 23(48%) selected ceftriaxone. Also, 8 (16%) selected vancomycin or clindamycin as an alternative for patients with a history of Ig E-mediated reaction to penicillin. Conversely, a majority of them had correctly selected vancomycin as a drug of choice in cases of MRSA colonization. Concerning the sources of knowledge regarding SAP administration, textbooks and articles were the most selected, since 36 (74%) had selected them. Table 2 shows the knowledge of respondents regarding varies aspects of SAP.

**Table 2:** Knowledge of respondents regarding varies aspects of SAP

Questions	Response rate (%)
Types of surgery that require SAP:	
Clean surgery involving the placement of prosthesis or implant.	41 (84)
Clean non-prosthetic procedure.	17 (35)
Clean-contaminated surgery	36 (74)
Contaminated surgery	40 (82)
Dirty surgery	28 (57)
Accordance to the ASHP guidelines about SAP:	
The 1st choice for SAP in Gastro-duodenal surgeries (GR: Cefazolin).	6 (13)
For patients with a history of Ig E-mediated reaction to penicillin (GR:	8 (16)
Vancomycin or clindamycin).	
For procedures in which gram-negative pathogens are common (GR:	12 (25)
Ciprofloxacin or Gentamycin).	
For SAP in patients with Appendectomy for uncomplicated appendicitis	35 (71)
(GR: Cefazolin + Metronidazole).	
For MRSA colonization (GR: Vancomycin).	40 (82)
Sources of knowledge regarding SAP administration	
Textbooks and articles	36 (74)
Knowledge from initial training	25 (51)
Antibiotic prophylaxis guidelines	12 (25)
Consultation with an infectious diseases physician	11 (22)
Internet or personal experience	31 (63)

On the subject of hospital surgical antibiotics prophylaxis guideline, 48 (98%) of the doctors have agreed that there is a need for a hospital guideline for surgical antibiotics prophylaxis. Additionally, 18 (37%) of them said that they would be extremely cooperative in the establishment of hospital guidelines. While nearly half of the participants 24 (49%) said that they will refuse to cooperate due to various reasons, including the workload 7 (14%) and the lack of hospital guidelines in the hospital 16 (33%). Likewise, all the participants believe that there is a need for a national surgical antibiotic prophylaxis guideline, and 19 (39%) said that they are going to extremely cooperate for preparing that guideline. Nevertheless, 27 (55%) of them said they will not cooperate in establishing a national guideline, 19 (39%) said that because there are no national hospital guidelines, and only 7 (14%) are going to refuse to cooperate due to the heavy workload.

About adherence of doctors practice with ASHP guidelines, only 10 (20%) uses mechanical bowel preparation (MBP) in addition to oral antibiotics in elective colorectal surgeries, whereas the majority 21 (43%) uses mechanical bowel preparation (MBP) only. Also, 17 (35%) administer surgical antibiotics prophylaxis 60 min before the surgery. Yet, more than half of the 27 (55%) administer SAP during the induction of anesthesia. Moreover, for antibiotics that require longer periods of admission before the surgeries like vancomycin and fluoroquinolones, 6 (13%) of the respondents say that they administer these antibiotics 120 min before the surgery. On the other hand, nearly half of the 21 (43%) said that during anesthesia induction is the perfect time to administer such types of antibiotics. 21 (43%) of the participants repeat the dose of SAP in conditions like excessive blood loss more than 1500 cc, and when the operation’s duration exceeds two half-lives of antibiotic. Table 3 demonstrates the adherence of doctors with ASHP guidelines. Additionally, correlation between the academic level of participants and their practice, and between their knowledge and practice, was not significant (*p*-value = 0.6), (p-value = 0.8), respectively. However, there was a significant correlation between doctors’ knowledge and their academic level (p-value = .02, CC = − 0.33).

**Table 3:** Adherence of participants to ASHP guidelines

Guideline recommendations	Adherence N (%)	Non-adherence N (%)
Mechanical bowl preparation:		
mechanical bowel preparation should be used in addition to oral antibiotics.	10 (20)	39 (80)
Timing to administer parental prophylactic antimicrobials:		
30–60 min prior to surgical procedure.	17 (35)	32 (65)
Timing to administer parental prophylactic antimicrobials that include vancomycin and fluoroquinolones:		
120 min before surgery.	6 (13)	43 (88)
Conditions in which SAP dose is repeated:		
procedures that exceed two half-lives of prophylactic antibiotic or cause more than 1500 mL of blood loss.	21 (43)	28 (57)
Extension of SAP after surgery:		
24 h after surgery.	26 (53)	23 (47)

## Discussion

The results of this study showed that there is a deficiency in doctors’ knowledge regarding surgical antibiotics prophylaxis, especially in regards to the first line of choice in various clinical scenarios. Nevertheless, their attitude towards the need for national and local hospital guidelines was satisfactory, even though some of them said that they will not cooperate due to various reasons. Moreover, there was no association between the respondents’ knowledge and their practice. However, they showed poor adherence to ASHP guidelines, particularly in areas related to timing of administration and mechanical bowel evacuation.

ASHP guidelines rank cefazolin as the first drug of choice for SAP in many surgeries [15], however in this study doctors’ choice was ceftriaxone. In an audit conducted by Elbur et al. in Sudan, and despite the availability of cefazolin in the hospital, doctors reported using cefuroxime as their first choice [20]. Similarly, doctors’ choices varied among different countries, cefazolin was selected first in Iran [21]. However, third and second-generation cephalosporins were doctors’ choices in Turkey and Jordan, respectively [22, 23]. Moreover, cefazolin was selected in Canada, and co-amoxiclav in England [24, 25]. These variations might be due to many factors including differences in local guidelines, personal experiences, studies settings, and medication availability.

It is recommended that fluoroquinolones or aminoglycosides to be used if there is a risk of contamination with gram-negative bacteria [15, 26]. In this study, only 25% of the participants selected these antibiotics. Similar results were found in a study from Iran, in which only 14% had adequate knowledge regarding this subject [21]. Furthermore, vancomycin usage as surgical prophylaxis has been recommended in cases of MRSA colonization [27]. 82% of doctors correctly selected vancomycin in these cases, compared to 54% in another study [21]. This might be due to the numerous encounters of Sudanese doctors with MRSA colonization. For instance, a study conducted in a Sudanese tertiary state hospital revealed that nearly half of Staph Aureus specimens isolated from surgical wounds were methicillin-resistant [22]. Additionally, 82% of doctors in this study reported that SAP is used in contaminated surgeries. These results are coherent with other study from Jordan, in which doctors said that SAP should be used in contaminated surgeries [23]. However, in this situation, the guidelines clearly identify the usage of antibiotics as treatment and not as prophylaxis [15, 26]. The majority of doctors in this study selected textbooks and articles as their major source of knowledge regarding SAP, this finding is similar to Al-Azzam et al. and Baniasadi et al. findings [23, 21].

Regarding the need for hospital and national SAP guidelines, doctors in this study showed a good attitude on the subject of establishing such guidelines. However, many of them said that they will not cooperate in preparing these guidelines due to the lack of other local and national guidelines. In contrast to this, doctors from other study had a good attitude towards the establishment of guidelines [21].

Only 35% of the study participants adhere to the practice of administering SAP 30–60 min before the surgery. Instead, the majority of them administer SAP during the induction of anesthesia, a method that has been proven to be less effective than the guidelines’ recommendations [26]. Another study from Sudan also reported similar results, in which only 9% of its participants were adherent with this recommendation [20]. Analogous results were also revealed by Beer et al. [24]. On the other hand, a majority 74% of Baniasadi et al. study participants were adherent with this guideline [21]. Repeating intraoperative administration of SAP is endorsed in cases of excessive blood loss more than 1500 cc or prolonged procedures [26]. In this study, only 43% of the participants were adherent to this recommendation. Harmoniously, only 40% of participant doctors from Iran adhere to this guideline [21].

This study had a limitation of a relatively small number of participants. However, it highlighted the lack of adequate knowledge and poor adherence to practice guidelines of surgeons regarding the topic of SAP. Adapting and preparing a national SAP guideline based on the epidemiology and susceptibility patterns of common pathogenic organisms is highly recommended. And institutionalization of such guidelines based on the specific data from every hospital is furthermore recommended. Also, nationwide studies are needed to identify the gaps in knowledge and practices of doctors. Furthermore, Implementation of these guidelines into practice is crucial, and thus, an institution-based training program about surgical antibiotics prophylaxis is extremely endorsed.

## Conclusion

The current results revealed that the knowledge and practice of surgeons concerning SAP had some inconsistencies with the available scientific evidence. Effective educational programs and compiling local and hospital guidelines by a group of surgeons, clinical pharmacologists, and infectious disease physicians may improve SAP prescription and decrease SSIs.

## Abbreviations

ASHP: American society of health system pharmacist
MBP: Mechanical bowel preparation
SAP: Surgical antibiotic prophylaxis
SSIs: Surgical site infections
